# Minimal Access Aortic Valve Surgery

**DOI:** 10.3390/jcdd10070281

**Published:** 2023-06-30

**Authors:** Bilal H. Kirmani, Enoch Akowuah

**Affiliations:** 1 Department of Cardiothoracic Surgery, Liverpool Heart and Chest Hospital, Liverpool L14 3PE, UK; 2 Cardiac Surgery, Faculty of Medical Sciences, Newcastle University, Newcastle upon Tyne NE2 4HH, UK; enoch.akowuah@nhs.net; 3 Academic Cardiovascular Unit, South Tees NHS Foundation Trust, Middlesbrough TS4 3BW, UK

**Keywords:** minimally invasive, aortic valve, surgery

## Abstract

Minimally invasive approaches to the aortic valve have been described since 1993, with great hopes that they would become universal and facilitate day-case cardiac surgery. The literature has shown that these procedures can be undertaken with equivalent mortality rates, similar operative times, comparable costs, and some benefits regarding hospital length of stay. The competing efforts of transcatheter aortic valve implantation for these same outcomes have provided an excellent range of treatment options for patients from cardiology teams. We describe the current state of the art, including technical considerations, caveats, and complications of minimal access aortic surgery and predict future directions in this space.

## 1. Introduction

Aortic valve disease is common, affecting 1 in 100 adults in the United States and some six million or so individuals worldwide [1,2,3]. A heterogeneous spectrum of conditions with different aetiologies and pathophysiologies can lead to valve stenosis, regurgitation, and endocarditis. The natural history of these conditions is progressive, with symptom progression from angina, breathlessness, or syncope eventually culminating in heart failure and death. In the western world, senile degenerative stenosis predominates, whilst elsewhere, rheumatic disease is more prevalent. The onset of symptoms is thought to mark an inflection point at which compensatory mechanisms are exhausted, and prognosis worsens quickly. Most patients with severe aortic stenosis will develop symptoms within five years, and event-free survival may be as low as 21% at two years [4,5]. Even with moderate aortic stenosis, progression leads to poor prognostic disease in 38% of patients within five years [6]. No medical treatments influence the natural history of aortic stenosis [7].

Treatment for aortic valve conditions, therefore, invariably consists of replacement of the diseased valve in 99% of cases [8]. While repair procedures have gained traction in some cases, replacement of the valve has remained the principal strategy since its inception in 1958. Outcomes have been excellent, with mortality from isolated, uncomplicated, and conventional aortic valve replacement consistently less than 1% [9].

Despite excellent outcomes for surgery, a 33% rate of surgical turn-down for patients over the age of 75 y in the Euro Heart Survey prompted a search for less invasive means of treatment for aortic valve disease [10]. The first percutaneous transcatheter aortic valve implantation (TAVI) in humans was performed in 2002 [11]. Over the last two decades, the technology has matured and increased in efficacy and scope, leading to increasingly liberal recommendations for its use in the guidelines [12]. Conversely, the 2017 European Society of Cardiology/European Association of Cardio-Thoracic Surgery guidelines did not reference minimal access approaches for valve surgery at all, and when updated in 2021, these approaches were still not discussed [13]. Transcatheter techniques, with a compellingly short recovery time even in highly comorbid patients, have been a driver reducing the invasiveness of conventional surgical approaches. Complications associated with TAVI, such as cerebrovascular embolic events, vascular complications, conduction disorders, and prosthetic valve dysfunction, are gradually being addressed, and they provide a good rationale to consider the known excellent pedigree of surgical valve replacement as a first-line treatment for the majority of patients requiring interventions.

Having both approaches available in the armamentarium of cardiology teams serves the needs of patients well. Nevertheless, there remains an appetite to marry the reduced invasiveness of TAVI with the meticulous decalcification and directly visualized seating of uncrimped valves. At the turn of the millennium, Chitwood predicted that 21st century cardiac surgery procedures would be performed minimally invasively as day-cases, with patients returning to normal activity within one or two weeks [14]. Nearly 25 years later, this belief has proven true for percutaneous interventions, but it remains an elusive goal in surgery.

Numerous systematic reviews, referenced through this paper, have previously documented defined outcomes for minimally invasive aortic valve surgery against the current standard of care. We do not replicate this format here but synthesize our experience and opinion as both practitioners and as authors of a Cochrane review on the subject. We have not systematically searched the literature but rather seek to fill the gaps with other existing systematic reviews. In this state-of-the-art review, we describe advances in surgical techniques that have improved the quality and outcomes of minimal access aortic valve surgery, and we present a vision for future direction.

## 2. Minimal Access Options

While the terms *minimal invasiveness* and *minimal access* are often used interchangeably, these overlapping philosophies can be disparate. In particular, the relative technical challenges of performing surgery through reduced access incisions can increase cardiopulmonary bypass and ischaemic surgical time, paradoxically increasing the invasiveness of the procedure. The invasiveness of cardiac surgery, some have posited, is as much contributed to by the deleterious effects of cardiopulmonary bypass as it is by the trauma of sternotomy, which is generally well tolerated [15,16]. Efforts to mitigate for both to make incremental gains have included a plethora of techniques and approaches, each with various advantages, disadvantages, and prerequisites. The most common are summarized here, and it is worth noting that there is a paucity of evidence to support preferring one approach over others [17].

### 2.1. Approach

#### 2.1.1. Hemi-Sternotomy

The hemi-sternotomy (also known as partial sternotomy or limited sternotomy) approach that is now the most common approach to minimally invasive aortic valve surgery was developed after initial work using mini-thoracotomy [18,19]. The rationale for this approach was to avoid the subcostal neurovascular bundles that can cause post-thoracotomy pain if retracted, as well as to improve access to the great vessels for central cannulation.

**Figure 1:**
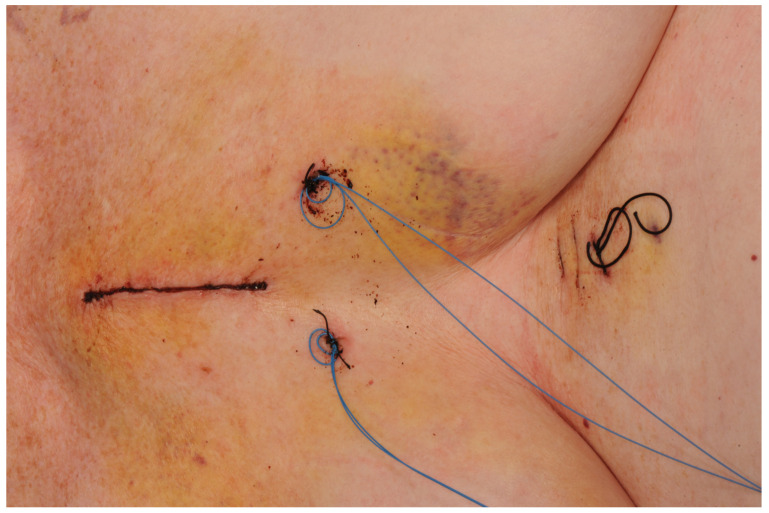
Hemisternotomy incision.

**Figure 2:**
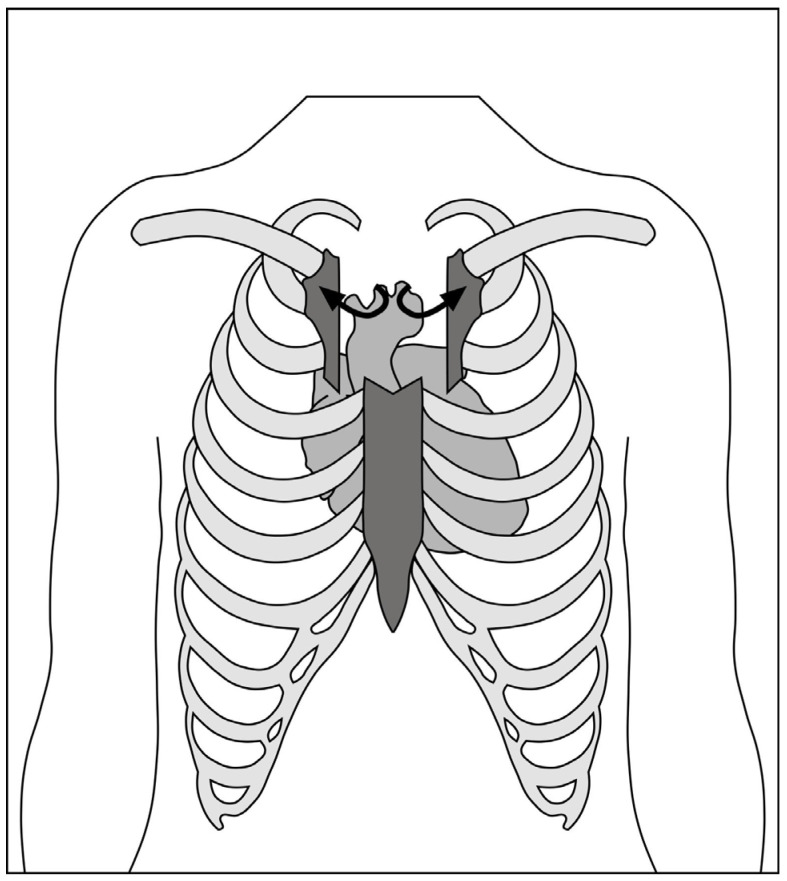
Schematic of chevron-shaped hemi-sternotomy into bilateral second intercostal spaces.

The skin incision (Figure 1) is made over the upper midline to access the aorta, with a J-shaped, L-shaped, or chevron sternotomy into the right, left, or bilateral intercostal spaces (Figure 2). The length of the sternotomy can vary and may be into third or fourth intercostal space. Accordingly, the length of the incision can also vary, with 5 cm being a typical lower limit. This modifiable access can be utilized to undertake not only aortic valve replacement but also surgery of the aortic root, ascending aorta, and hemi-arch [20].

The upper hemi-sternotomy approach can provide sufficient access to perform complete central cannulation with conventional instruments and cannulae, if desired, although modifications and training to facilitate surgery have been well described [21,22]. A 2017 Cochrane review of randomized, controlled trials comparing full sternotomy to hemi-sternotomy found that, across 10 included studies, the evidence was of low certainty due to biases and small sample sizes [23]. For most major outcomes, including peri-operative mortality, pain, or quality of life, there was no significant difference. Blood loss was slightly lower with hemi-sternotomy compared to full sternotomy (mean difference 153 mL lower compared to 400 mL), and index admission costs were also higher (a mean difference of £1190 (~$1470) more for hemi-sternotomy). Following the publication of the Cochrane review, the publication of two well-designed trials performed in the UK, MAVRIC and Mini-Stern, prompted a revised review of the literature.

*The MAVRIC Trial*

The Manubrium-limited Mini-sternotomy versus Conventional Sternotomy for Aortic Valve Replacement (MAVRIC) trial was a single centre, randomized, controlled trial comparing patients undergoing AVR via manubrium-limited mini-sternotomy with an AVR via conventional sternotomy group [24,25].

Patients in the intervention arm received a manubrium-limited mini-sternotomy, performed using a 5- to 7-cm midline skin incision dividing the manubrium from the sternal notch to 1 cm below the manubrium–sternal junction. Cardiopulmonary bypass (CPB) was established with an ascending aortic cannula and percutaneous femoral venous cannulation. Those in the usual care arm received conventional median sternotomy.

The primary outcome was the proportion of patients who received a red cell transfusion postoperatively and within seven days of AVR surgery.

The trial reported that mini-sternotomy was not found to be superior to conventional sternotomy with respect to red cell transfusion requirements within seven days of surgery. The proportion of patients receiving red cell transfusion was 23 of 135 in both groups (odds ratio 1.0 (95% CI: 0.5, 2.0) and risk difference of 0.0 (95% CI: −0.1, 0.1)). However, secondary endpoints showed that there was a statistically significant difference with respect to transfusion volumes of non-red cell blood components. Mini-sternotomy also resulted in a relative reduction in chest drain losses; however, greater blood loss in the conventional group did not translate into red cell transfusions. Patients in the mini-sternotomy group had significantly longer bypass and cross clamp times and worse lung function at four days post-surgery. Lung function at 12 weeks and adverse event rates were otherwise not different between the groups.

*Mini-Stern Trial*

Mini-Stern was a multi-centre, open-label, pragmatic, randomized, controlled trial with primary end points of postoperative length of hospital stay and time to fitness for discharge. In total, 222 patients were randomized, and it was found that mini-sternotomy patients had no difference in length of stay (median 7 (interquartile range (IQR 6–10) vs. 7 (IQR 6–10), *p* = 0.692) and no difference in time to fitness for discharge (median 5 (IQR 5–10) vs. median 6 (IQR 5–9), *p* = 0.560). Mini-sternotomy was £1719 more expensive per patient compared to full sternotomy in the first year following surgery. There was no significant difference in EQ-5D-based quality-adjusted life years (QALYs); therefore, at a willingness to pay threshold of £20,000 per QALY, there was only a 3.7% chance that mini-sternotomy was cost-effective.

*Current meta-analysis*

As of August 2021, there were 15 published randomized, controlled trials of 1395 participants comparing hemisternotomy with full median sternotomy due to be published shortly as an update of the previous Cochrane review. Countries of origin included Austria, the Czech Republic, Spain, Italy, Germany, France, Egypt, Russia, Sweden, Serbia, and the United Kingdom [26,27,28,29,30,31,32,33,34,35,36,37,38,39]. All but one were single-centre studies of elective, isolated aortic valve replacements, typically excluding patients with poor left ventricular function. The European studies were predominantly focused on patients with degenerative heart valve disease and therefore an older population, whereas the study from Egypt included younger patients, presumably with rheumatic heart disease. The surgical strategy was broadly similar between studies, with aortic arterial cannulation and either central or femoral venous cannulation. Venting strategies—often considered a source of concern in minimally invasive aortic valve replacement due to potential compromises in bloodless field, de-airing, or left ventricular decompression—were variable.

The risk ratio for peri-operative mortality was 0.93 (95% confidence interval [CI] 0.45–1.94, in 10 studies, but with low certainty). The mean difference in cardiopulmonary bypass time was 10.7 min longer with ministernotomy (95% CI 3.3–18.0 in 10 studies, but with very low certainty). The mean difference in aortic cross-clamp time was 6.1 min longer with ministernotomy (95% CI 0.8–11.3 across 12 studies but also with very low certainty). Even in this context of uncertainty, these mean differences in extra-corporeal circulation and ischaemic times are likely to have had very little clinical impact. Exclusion of one trial [31] in which rapid deployment valves were used to facilitate expedient surgery in the minimally invasive arm only and not in the full sternotomy arm did not impact the finding of the small increases in bypass and cross-clamp times.

There was a modest reduction in length of stay in patients undergoing mini-sternotomy (mean difference 1.1 days less (95% CI −1.9 to −0.3 days across 11 studies with very low certainty). It is important to note that, for most of these studies, trial protocols were not published *a priori* or at all. Outcome measures, such as hospital length of stay, which might be contingent on different criteria between studies and which was considered at high risk for bias from blinding, were not directly comparable. Similar issues arose with intensive care length of stay, which, in studies at low risk of bias, was marginally shorter with minimally invasive surgery (−0.45 days, 95% CI −0.84 to −0.06).

In-hospital pain assessments were also no different between minimally invasive and full sternotomy approaches (standardized mean difference −0.19 for minimally invasive, 95% CI −0.43 to 0.04 with low certainty). Equally, there was no difference in quality of life measures after discharge from hospital (mean difference 0.03, 95% CI 0.00 to 0.06 across 4 studies with low certainty). Finally, pulmonary function tests—considered a surrogate marker for comfort and capability following disruption of the thoracic skeleton—were also minimally different between the two approaches (mean difference 2.1% higher with mini-sternotomy, 95% CI 0.74 to 3.41).

This new meta-analysis would appear to demonstrate that ministernotomy aortic valve replacement is as safe as full-sternotomy surgery but with few of the anticipated advantages regarding pain, quality of life, or breathing that have been cited as reasons to perform minimally invasive surgery. Length of stay in the hospital and in the intensive care unit was modestly better with minimally invasive surgery but at an increased cost over standard of care.

#### 2.1.2. Right Anterior Mini-Thoracotomy

**Figure 3:**
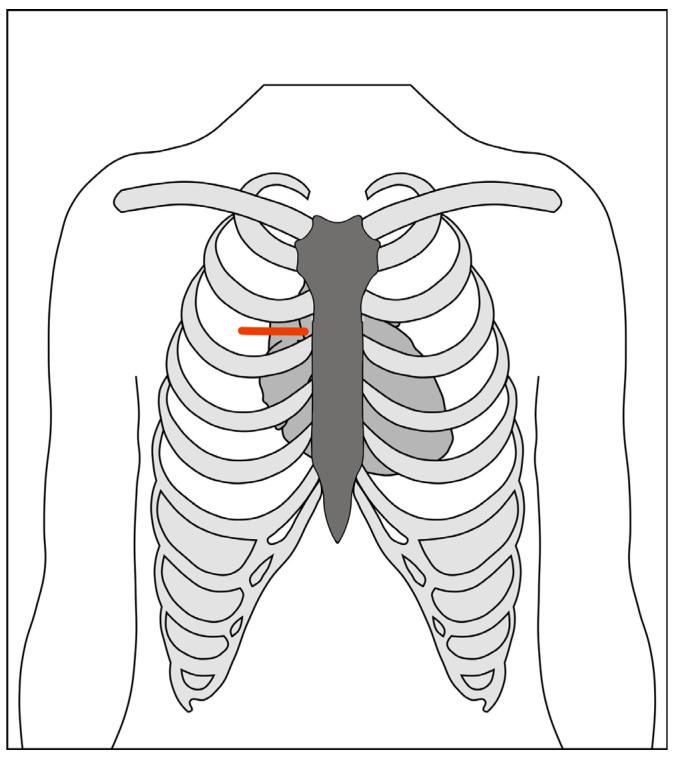
Schematic of right anterior minithoracotomy through second intercostal space.

The right anterior mini-thoracotomy (RAT) approach, using central cannulation, was first described by Rao and Kumar in 1993 [40]. Cosgrove and Sabik first applied the term “minimally invasive” to a 10-cm thoracotomy with excision of two costal cartilages, utilizing a femoral bypass [41]. The current technique accesses the mediastinum through the second intercostal space (Figure 3). The main advantage of this approach is the absolute preservation of sternal integrity and the absence of lateral traction, similar to the parasternal approach described by Cohn [42]. The corollary to these benefits is the need for femoral cannulation because of limited access, necessitating a second incision in the groin and retrograde perfusion, which may offset the cosmetic advantages, quality of life, and satisfaction with minimally invasive surgery [43]. A rate of 1% of non-union of the transected ribs may also cause intractable post-thoracotomy pain. 

Initially, meta-analyses found RAT to have shorter hospital stays compared to hemi-sternotomy [44,45], despite higher rates of bleeding, transfusion, and conversion to full sternotomy. A more recent comprehensive network meta-analysis of propensity matched and randomized studies compared full sternotomy, hemi-sternotomy, and right anterior mini-thoracotomy [46]. This study found no differences in surgical time, hospital length of stay, or ventilation time between the two minimally invasive approaches. As with previous meta-analyses, return to the theatre for bleeding was more common with right anterior mini-thoracotomy than with hemi-sternotomy (RR 1.65, 95% CI 1.18–2.30, *p* = 0.003).

The network meta-analysis included 42 studies, including 29 propensity matched studies and 13 randomized, controlled trials. The majority compared median sternotomy to hemisternotomy, with nine comparing RAT to full sternotomy and two comparing RAT to hemisternotomy. Notably, only one randomized study evaluated RAT, so much of the evidence was observational, albeit adjusted with propensity matched techniques. Again, the majority of the included studies were European in origin, and the methodological quality was fraught with uncertainty. That this study identified a significant peri-operative mortality advantage of hemi-sternotomy over median sternotomy (relative risk (RR) 0.60, 95% CI 0.41–0.90) or RAT (RR 0.50, 95% CI 0.27–0.97) should be interpreted with caution. A meta-analysis of randomized trials with didactic scrutiny of methodology and quality found no difference in mortality between hemi-sternotomy and full-sternotomy [23]. There also exists little rationale to explain why hemi-sternotomy should reduce mortality; indeed, all plausible mechanisms for a mortality difference between minimally invasive aortic valve replacement techniques might suggest that minimal access approaches are at *higher* risk than conventional surgery for peri-operative mortality. Therefore, it is our interpretation that, while the network meta-analysis favours hemi-sternotomy as the access of choice for aortic valve surgery (over both full sternotomy and right anterior mini-thoracotomy), the evidence to support this suggestion is scant.

The authors of a recent consensus statement [47] acknowledged the increased technical challenge of a right anterior mini-thoracotomy approach. Additional equipment, such as thoracosopes, long-handled instruments, and soft tissue retractors, require specific training. Cost comparisons vary across publications. In some, the need for additional equipment and consumables increases the costs of RAT to up to US$4209 higher than full sternotomy, compared to US$290 more for hemisternotomy [48,49]. In others, the costs of RAT have been between US$1891 to US$3887 lower when propensity matched across real-world registry data [50,51].

Right anterolateral mini-thoracotomy (in the third intercostal space) can be used to perform multiple valve replacements, including of the aortic, mitral, and tricuspid valves [52,53,54]. Table 1 summarises some of the characteristics of the different approaches.

#### 2.1.3. Hybrid Approach (Sternotomy + Transcatheter Aortic Valve Implantation)

While the hybrid approach is not strictly minimal access and ostensibly not minimally invasive, patients not fit for conventional aortic valve replacement who also require coronary artery revascularization may benefit from lower invasiveness using a hybrid approach. Sternotomy may be performed to undertake off-pump coronary artery bypass grafting, with concomitant trans-aortic or trans-femoral TAVI. The procedure avoids the sequelae of cardiopulmonary bypass in patients who may not tolerate it while still allowing for complete revascularization and management of aortic stenosis [55,56,57].

### 2.2. Cannulation and Cardiopulmonary Bypass

#### 2.2.1. Central

Hemi-sternotomy to the third or fourth space usually provides sufficient access to the aorta and right atrium to cannulate for arterial inflow and venous drainage centrally. The right superior pulmonary vein may or may not be accessible with this approach. Standard cannulae may be used, although to aid retraction of the pipes out of the field of view, some surgeons prefer angled venous pipes or a flat/low-profile cannula. Vacuum assistance can also help to improve the venous drainage.

#### 2.2.2. Peripheral

Where the incision limits the access to the aorta, there may only be space in the surgical field for the cross-clamp, cardioplegia site, and aortotomy. In this case, peripheral cannulation, typically at the femoral artery, is utilized. Percutaneous methods are possible, but direct surgical access is the more commonly performed approach. Complications arising from groin cannulation may occur in 10.8% of cases, with seromas in up to 5% [48]. Percutaneous cannulation might mitigate some of this risk.

#### 2.2.3. Hypothermia and Systemic Hyperkalaemia

In the context of re-operative minimal access surgery, systemic hypothermia to 20 °C along with systemic hyperkalaemia at 7 mmol/L can support myocardial protection in the presence of patent left internal mammary bypass grafts [58]. This process requires ultrafiltration on cardiopulmonary bypass following cardiac reperfusion. Retrograde flooding of the field from the left coronary ostium in the presence of an unclamped left internal mammary graft can be mitigated using intermittent circulatory arrest to facilitate suture placement or peri-operative percutaneous balloon occlusion of the left internal mammary by cardiology.

#### 2.2.4. Venting (and Imaging)

Reduced access to the left ventricle for inspection or manual decompression mandates the use of a trans-oesophageal echo during minimally invasive aortic valve surgery. Whereas this procedure may be omitted in special circumstances in conventional sternotomy, such as oesophagectomy or oesophageal stricture, the risks are much greater in minimal access surgery, during which the left ventricle cannot be directly assessed.

Dependent on the level of access, left ventricular venting can be achieved in different ways including:

- The right superior pulmonary vein;
- The pulmonary artery;
- Trans-aortically.

Access to the pulmonary veins is typically not possible except in larger partial sternotomy approaches (i.e., fourth intercostal space). The pulmonary artery is usually easily accessible, although the visualization decreases considerably once the cross-clamp is removed, and the efficacy of venting is variable. Access further deteriorates off cardiopulmonary bypass; therefore, it is imperative to achieve haemostasis of the vent site early. The trans-aortic vent approach is usually sufficient to provide a bloodless field for surgery, but it is limited should venting be required during reperfusion since the left ventricle cannot be reached manually.

### 2.3. Adjuncts

#### 2.3.1. Rapid Deployment Valves

Sutureless and rapid deployment valves have been described as obvious companions for minimal access aortic valve replacement to compensate for the increased cardiopulmonary bypass and cross-clamp times that are otherwise seen [31,59]. These procedure typically have excellent haemodynamic profiles, but they have a shorter pedigree and may, with their similarities to transcatheter valves, have a greater propensity towards structural valve deterioration.

#### 2.3.2. Automatic Suture/Knotting Devices

Automatic suture placement or knotting devices reduce invasiveness in minimal access valve surgery by expediting valve implantation and therefore reducing cross-clamp and cardiopulmonary bypass times [60]. The use of the Cor-Knot^®^ device (LSI Solutions, Victor, NY, USA) for valve surgery was noted to also lead to more reproducible valve implantation with lower rates of paravalular leakage [61]. The same manufacturer has also released an automated annular suturing device to expedite this stage of valve implantation, but there are not yet any published series showing that it is effective.

#### 2.3.3. Transvenous Pacing, Cannulation, and Venting

A variety of options exist for percutaneous support of cardiopulmonary bypass from the right jugular. These options include:

- Transvenous pacing can be floated using an inflatable balloon tip into the right ventricle for endocardial pacing if the anterior right ventricle is not accessible;
- Coronary sinus cannulation via the right internal jugular was previously possible using the Proplege device (Edwards LifeSciences, Irvin, CA, USA). but as the strategies for anterograde cardioplegia alone showed good efficacy, it is no longer available;
- Pulmonary artery venting through a percutaneously floated catheter has again lost favour, as trans-aortic and direct pulmonary artery or pulmonary vein venting have been shown to be efficacious and safe.

#### 2.3.4. Thoracoscopes

Direct visualization of the aorta (sufficient to apply a cross-clamp), aortic valve, and aortic annulus is possible through both hemi-sternotomy and right anterior mini-thoracotomy approaches. Thoracoscopes can also be utilized to improve the light and view for areas that might be more difficult to see due to an overhanging thoracic cage. Use of scopes for lighting and/or transmitted video images may, however, limit access for instruments in some cases. This limitation can in part be mitigated using other adjuncts, such as automatic suture placement or knotting devices [47].

#### 2.3.5. Robot Assistance

Robotic aortic valve replacement has been attempted since 2004 [62], with a renewed interest in recent years [63]. In place of either hemi-sternotomy or right anterior mini-thoracotomy, a right *lateral* mini-thoracotomy is utilized as the working port, along with four arms, sparing division of the costal cartilage or sacrifice of the right internal mammary artery. This procedure has the advantage of an intact thoracic skeleton, superior visualization, and virtually unrestricted range of movement in the working space but with a steep learning curve, high capital investment costs, and ongoing consumable expenditures.

### 2.4. Special Circumstances

#### 2.4.1. Concomitant Procedures

As minimal access incisions have gained favour, indications for procedures amenable to this approach have expanded. The hemi-sternotomy approach has been successfully utilized for aortovascular procedures, including valve sparing root replacement [64].

#### 2.4.2. Re-Do Procedures

Previous sternotomy, even in the presence of patent coronary artery bypass grafts, is not a contraindication for a minimally invasive approach. These procedures can be performed with low-conversion rates of 2.6% [58]. Minimization of the dissection and mobilization of the heart mean that bleeding complications are low. Technical challenges from the presence of patent grafts may require alternative strategies for cardioplegic arrest (including systemic hyperkalaemia, deeper hypothermia, and brief periods of circulatory arrest). However, since transcatheter techniques for aortic valve replacement have improved in safety and reliability, they are often considered the first choice in such anatomical conditions, which can be difficult or hostile for conventional surgery.

## 3. Minimal Access Pre-Operative Planning/Setup

Additional assessment and preparation are required for minimal access aortic valve surgery. These requirements vary depending on the approach adopted, and not all practitioners employ all these steps. Indeed, some proponents claim that minimal-access aortic valve replacement can be offered to all comers with no patient selection, whereas most would agree that some absolute and relative contraindications exist for each technique. The specific instructions for each technique are beyond the scope of this review.

- A CT scan pre-operatively can allow for assessment of the position of the aorta relative to the incision planned. If peripheral cannulation is intended, CT can also determine whether the femoral vessels are of an adequate calibre and the descending aorta is free of mobile atheroma that may preclude retrograde perfusion.
- Short-acting anaesthetic drugs should be considered to facilitate early extubation and enhanced recovery.
- A sheath in the right internal jugular vein can be introduced at the time of induction of anaesthesia if the usual access limits epicardial pacing wires.
- A bag of saline behind the shoulder blades can elevate and expand the chest, providing an improved approach to the mediastinum.
- External defibrillator pads are required to cardiovert ventricular fibrillation, as internal paddles cannot be applied to the heart.
- Trans-oesophageal echocardiography is mandated for minimally invasive aortic valve surgery, as the direct visualization of the right and left ventricular function is impaired.
- A double lumen tube or bronchial blocker for selective ventilation of the right lung can facilitate the early learning curve [65].
- Carbon dioxide field flooding can aid in de-airing at the end of the case, when cardiac massage is not possible and venting is limited. Passive and limited active de-airing can therefore be supplemented with displacement of air in the cardiac chambers with highly soluble CO_2_.

## 4. Outcomes

It is unlikely that minimally invasive methods of aortic valve surgery would have been permitted to develop if peri-operative mortality was not equivalent to that with conventional full sternotomy. While the excellent current outcomes for isolated aortic valve replacement mean that most series have not had sufficient power to demonstrate a difference, it is apparent that there is no excess mortality.

Some credence has been placed on minimal access surgery to therefore also improve outcomes in other areas and justify the technical challenges and increased costs.

### 4.1. Pain

For studies that examined post-operative pain scores between minimal-access and full-sternotomy surgery, there has been considerable bias in the reporting [23]. Protocolized analgesic pathways were seldom cited, and blinding was only utilized in one trial. The timing of pain score assessments also varied considerably between studies. The overall assessment following the review was that minimal access aortic valve surgery did not reduce pain compared to sternotomy.

### 4.2. Respiratory Mechanics

The impact of full median sternotomy on respiratory function following surgery is often cited as a reason to favour minimal access approaches. While there might be small differences in peri-operative lung function parameters between non-sternotomy and full-sternotomy approaches, the main advantage to respiratory mechanics is in the time to return to baseline. For partial sternotomy, this period was one month, whereas for full sternotomy, it was up to three months [66].

### 4.3. Quality of Life

Five randomized, controlled trials studied quality of life following surgery. Between 6 and 12 weeks following surgery, there was no difference in the quality of life scores between hemi-sternotomy and full-sternotomy [23].

### 4.4. Complications

While numerous reviews have compared minimal-access and full-sternotomy aortic valve replacement for rates of major complications, such as mortality, stroke, bleeding, etc., some complications are particular to minimal access surgery. Poor visualization or access can increase the chances of iatrogenic injury (especially to great vessels, as well as right ventricular and aortotomy bleeding) and can also increase the likelihood that this injury cannot be controlled. Conversion from minimal-access to full-sternotomy is usually sufficient to resolve the poor exposure and occurs in between 0.8% and 8.0% of cases depending on centre experience, with many reports citing 3–4% conversion rates [67].

## 5. Discussion

Chitwood’s prediction (or aspiration) that cardiac surgery would culminate in routine minimally invasive approaches that would emulate general surgery’s day-case model still seem very distant a quarter of century on. Large series initially expounded by forerunners, such as Cohn, Mohr, Roselli, Solinas, Glauber and Lamelas, have inspired considerable growth, but the procedure is far from the standard approach [68]. The benefits of minimal access surgery have not been realized as predicted, and in the intervening decades, transcatheter techniques have improved and expanded their remit to low-risk patients [69]. The cost-effectiveness of percutaneous methods has also improved such that TAVI procedures are now competitive against surgical valves [70].

In the absence of demonstrable superiority, there is therefore more incentive than ever to propagate minimal access approaches to aortic valve replacement. Whereas these procedures can be performed with minimal or no additional equipment, at a similar cost, and with no increase in complications, the new standard should surely be the procedure with the same outcomes, same costs… and a smaller incision? Cosmetic superiority, in addition to non-inferiority for all other key metrics, is a convincing argument for minimal access aortic valve surgery, especially when driven by patient preference.

The current state of the art in minimal access aortic valve surgery is advanced. Even in groups such as octogenarians, re-operations, and root/ascending aorta replacements, the results for minimal access aortic surgery can be excellent [71]. The procedure is reproducible and can be undertaken by surgeons in training without compromising patient safety [72]. Peri-operative and one-year mortality rates are no different in a real-world setting, and the length of stay is typically reduced using a minimal-access approach [73].

As transcatheter aortic valve implantation also gathers momentum, gaining approval for increasingly lower-risk patient groups, there is a need to ensure that patients are provided with procedures with good pedigrees. Surgeons seeking to compensate for the increased operative times of minimally invasive aortic valve replacement may turn to sutureless aortic valves, drawing parallels with transcatheter valves. Whereas TAVI valves have higher rates of paravalvular leakage, pacemaker implantation, and vascular complications [74], sutureless valves can offer the advantage of full decalcification and placement under direct vision. Whether these benefits offset the need for thoracic cage disruption, cardiopulmonary bypass, and cross-clamping remains to be seen, particularly in small or calcified roots.

Further research is also still required to elucidate the differences between hemi-sternotomy and right anterior minithoracotomy. The former remains the more accessible minimally invasive approach, with familiar setup, angles, and equipment to those used for training in conventional full sternotomy. Proponents of the right anterior mini-thoracotomy, however, will argue that, if a partial-sternotomy is superior to a full-sternotomy, it stands to reason that no sternotomy should be better still. However, it remains to be compellingly proven that the additional challenges of RAT do not neutralize the benefits provided by maintaining the sternum fully intact. Current syntheses of the existing literature have been disparate in their conclusions.

Future directions for this procedure involve ensuring that it is, indeed, developed as the new standard of care for aortic valve procedures. Once this principle is accepted universally, the process of reducing invasiveness, as well as access, can be developed. Enhanced recovery after surgery (ERAS) has been slow to be adopted in cardiac surgery in general and much less in minimal access cardiac surgery. Protocols are needed for patients with reduced incisions that differ from those used for conventional surgery, which might meaningfully engage and utilize the operative advantages to expedite the patient journey more effectively [75].

## 6. Conclusions

Minimal access aortic valve surgery is at a watershed moment at which it could plausibly become the new standard of care for aortic valve disease, as laparoscopic procedures have become for general surgery. Unlike the laparotomy versus laparoscopy analogy, however, minimally invasive aortic valve surgery appears to provide few or small benefits over open surgery, so is strongly driven by cosmetic considerations. Economic viability will need to be demonstrated for minimal access to become the default approach, but moreover, a surgical appetite must grow for non-inferior procedures in which the benefits are still being developed and demonstrated.

## Figures and Tables

**Table 1:** Advantages and disadvantages of aortic valve surgery approaches.

	Full Sternotomy	Hemi-Sternotomy	Right Anterior Minithoracotomy
Access	Unfettered view of whole mediastinum and whole heart	Good access to aortic root, limited to whole heart	Most challenging view
Sternal disruption	Whole sternum	To 2nd–4th intercostal spaces unilaterally or bilaterally	None, although costal cartilages are sometimes divided (may include right mammary artery ligation)
Cannulation	Full central	Variable—from full central to aortic arterial only	Typically requires peripheral cannulation
Instruments	Standard cardiac	Variable—can be standard or long-handled	Typically requires long-handled
Technical difficulty	Baseline	Learning curve easily traversed, including for trainee surgeons	Accepted to be technically challenging
Adjuncts Required	None	Variable—possible with standard equipment. Facilitated by rapid deployment valves, suture placement devices, and knot-tying devices	Facilitated by rapid deployment valves, suture placement devices, and knot-tying devices; Light source advantageous; Single lung ventilation.
Benefits (from most recent meta-analyses) *		Reduced intensive care and hospital length of stay; Reduced ventilation time	Reduced hospital length of stay; Reduced ventilation time; Lower stroke rate; Lower pacemaker rate
Risks *		Increased operative time; Increased costs	Increased operative time; Increased costs (including vs. ministernotomy); Lung herniation

* compared to median sternotomy unless stated otherwise.

## Data Availability

Not applicable.
